# Rapid high-resolution volumetric imaging via laser ablation delayering and confocal imaging

**DOI:** 10.1038/s41598-022-16519-2

**Published:** 2022-07-19

**Authors:** Adrian Phoulady, Nicholas May, Hongbin Choi, Yara Suleiman, Sina Shahbazmohamadi, Pouya Tavousi

**Affiliations:** University of Connecticut, Storrs, CT USA

**Keywords:** Engineering, Electrical and electronic engineering

## Abstract

Acquiring detailed 3D images of samples is needed for conducting thorough investigations in a wide range of applications. Doing so using nondestructive methods such as X-ray computed tomography (X-ray CT) has resolution limitations. Destructive methods, which work based on consecutive delayering and imaging of the sample, face a tradeoff between throughput and resolution. Using focused ion beam (FIB) for delayering, although high precision, is low throughput. On the other hand, mechanical methods that can offer fast delayering, are low precision and may put the sample integrity at risk. Herein, we propose to use femtosecond laser ablation as a delayering method in combination with optical and confocal microscopy as the imaging technique for performing rapid 3D imaging. The use of confocal microscopy provides several advantages. First, it eliminates the 3D image distortion resulting from non-flat layers, caused by the difference in laser ablation rate of different materials. It further allows layer height variations to be maintained within a small range. Finally, it enables material characterization based on the processing of material ablation rate at different locations. The proposed method is applied on a printed circuit board (PCB), and the results are validated and compared with the X-ray CT image of the PCB part.

## Introduction

Acquiring detailed 3D images of samples is needed for conducting thorough investigations in a wide range of applications, including but not limited to inspection, failure analysis, and reverse engineering^1,2^. Depending on the length scale and feature size of interest, different methods can be used for acquiring a 3D image. X-ray computed tomography (X-ray CT) can provide a fairly rapid, nondestructive solution for acquiring the 3D image of the sample, if feature sizes of down to ~ 1 μm are of interest, dosing is not an issue, and the X-ray absorption level of each region of the sample is sufficient for a follow-up analysis^3^. On the other hand, for resolutions down to sub-10 nm, one can use a repetitive delayering/imaging strategy, where a scanning electron microscope (SEM) or helium ion microscope (HIM) are used for imaging the layers of the sample, and methods such as focused ion beam (FIB) and knife technology (more suitable for biological applications) are used for removing thin layers of the sample to expose the buried layer for imaging^4,5^. These 2D images are then stacked to obtain a 3D tomographic image. Although technically, this method can be used for samples of arbitrary size, the low throughput of the process practically prohibits obtaining 3D images of large regions of interest (ROIs)^6^. The throughput issue often enforces the researcher to scout only a tiny region of the sample. As a result, the context of the entire sample is potentially lost. The throughput issue exists in imaging as well as the delayering process. Given that many studies need a resolution that is in the midrange of what is offered by X-ray and SEM, optical microscopy can potentially offer an alternative to the low throughput SEM and HIM, while providing better resolution and information content about the different regions of the sample of interest than X-ray. Applying a delayering process that is comparable, both in throughput and accuracy, with optical imaging, is challenging. The use of FIB for delayering would defeat the purpose, because of its low throughput.

There are alternatives to traditional FIBs, such as high current, gas-assisted, or plasma source FIBs^7–9^. However, these modalities sacrifice resolution for speed and still take days to remove cubic millimeters of material. Other common challenges of FIB include high cost and need for vacuum, curtaining effects^10^, and charging artifacts^11^ for nonconductive materials. On the other hand, other existing delayering techniques that potentially could have higher throughputs have precision and controllability issues. Mechanical grinding/polishing offers a relatively rough surface of the final polish and a poor vertical resolution (i.e., the distance between layers). Another major drawback of mechanical methods is the thermal and mechanical stress that is introduced to the sample^12^, which in some cases is detrimental to the sample’s integrity. Given that, in many applications, only one instance of the sample is at hand, this could result in losing the sample available for analysis. Further, to keep the sample intact during grinding, often an additional step, namely encapsulation in rigid material (e.g., epoxy), is necessary^13^. Chemical etching has controllability, quality, and hazard disadvantages in addition to the risk of losing the only available sample. In addition, for both cases of mechanical and chemical etching, extensive trial-and-error is needed for each specific case.

Femtosecond lasers, which have been shown to cause minimal to zero heat-affected zones (HAZs) can be considered as an alternative delayering method to the conventional mechanical and FIB methods^14^. Laser significantly outperforms FIB in terms of throughputs (offering 4–6 orders of magnitude faster material removal rates). Table 1 outlines the time needed to remove 0.3 mm^3^ of Platinum using FIB and laser.

**Table 1:** Comparison of the removal rates of the FIB and the femtosecond pulsed laser used in this work. The same material (here, Platinum) has undergone both the FIB and the laser processes.

Method	FIB	Laser	Laser w/GIS
Platinum ablation rate	0.97 µm^3^/s	4 × 10^4^ µm^3^/s	1.6 × 10^5^ µm^3^/s
Time for removing 0.3 mm^3^	1 year	11 min	17 s

In addition, compared to mechanical methods, lasers do not encompass tedious sample preparation steps, they do not endanger the integrity of the sample, and they could be material agnostic. A review of the application of femtosecond lasers for processing different materials is provided in Ref.^15^. A comprehensive study of the effect of different laser parameters such as wavelength, pulse duration and fluence, and temporal distribution of the laser pulses on the quality and throughput of the laser-enabled machining process has been provided in Ref.^16^. Thermal and non-thermal ablation mechanisms in crystalline silicon by femtosecond laser pulses are studied in Ref.^17^. A common challenge of femtosecond lasers, namely the trade-off between the use of high energy for achieving an efficient ablation rate and undesired phenomena such as saturation, shielding, and collateral damage from heat accumulation, can be addressed through the use of the ablation cooling technique, where high laser repetition rates are used to remove the heated material before the heat formed by the prior laser pulses can diffuse to the surrounding regions^18^.

**Figure 1:**
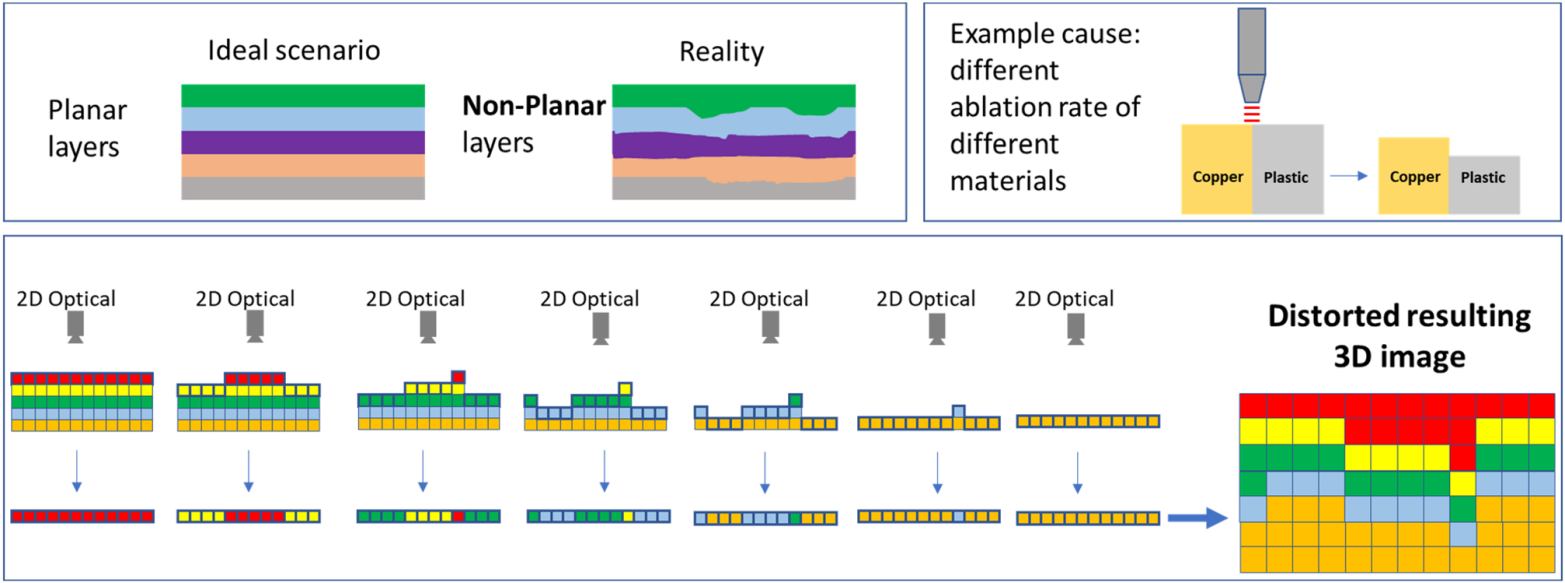
The challenge of stacking 2D images for obtaining a 3D image: due to nonplanarity of the 2D layers, the resulting 3D image will be distorted.

Delayering with lasers to conduct 3D volumetric imaging, however, faces a fundamental challenge. In conventional 3D volumetric imaging, it is assumed that the exposed layer of the sample, at each step, has a flat topography, an assumption that is used when reconstructing the 3D image by stacking the 2D layers. Nevertheless, due to the differences in the laser ablation rate of different materials, achieving flat cuts, which are the basis of acquiring tomographic images in the conventional approach, faces challenges. Differences in the interaction of the laser beam with different chemical compositions in a multi-material sample may lead to a drastically different depth of cut across different regions of the sample. Such an effect can drastically distort the final 3D image, as schematically depicted in Fig. 1. Further, even having access to and applying a priori knowledge about the material composition distribution across the sample of interest cannot fully address the layer non-flatness issue.

In this work, we propose to leverage confocal microscopy to address the challenges of obtaining flat layers. In fact, for obtaining the final 3D image, instead of relying on images of 2D layers that may be non-flat, this method will integrate the confocal microscopy to acquire a height map of the exposed layer of the sample, in addition to its optical image, at each step throughout the process and will apply that in the 3D image reconstruction. The height map will also be used for planning the follow-up lasering steps. That is, regions that have been cut deeper than a certain threshold will be excluded from the upcoming lasering step, in an effort, so-called masking, to maintain the height variation across the region of interest within a set limit. A benefit of doing so is to ensure that the confocal images of the exposed layers can capture the entire region of interest.

Another major challenge of using lasers is the redeposition of laser-ablated material^19^ onto the machined surface which could significantly impact the final 3D image quality. Although the use of air blow and vacuum suction can mitigate this, such methods are not 100% effective. In this work, we propose the use of targeted gas cleaning to address this issue.

A powerful byproduct of our method is automatic material characterization and 3D image segmentation. The proposed method uses the difference in the ablation rate of different materials when similar lasering parameters are practiced. Finally, the embedded computer vision algorithms ensure a fully automated process.

## Materials and methods

### Creating a 3D image from non-flat 2D images

**Figure 2:**
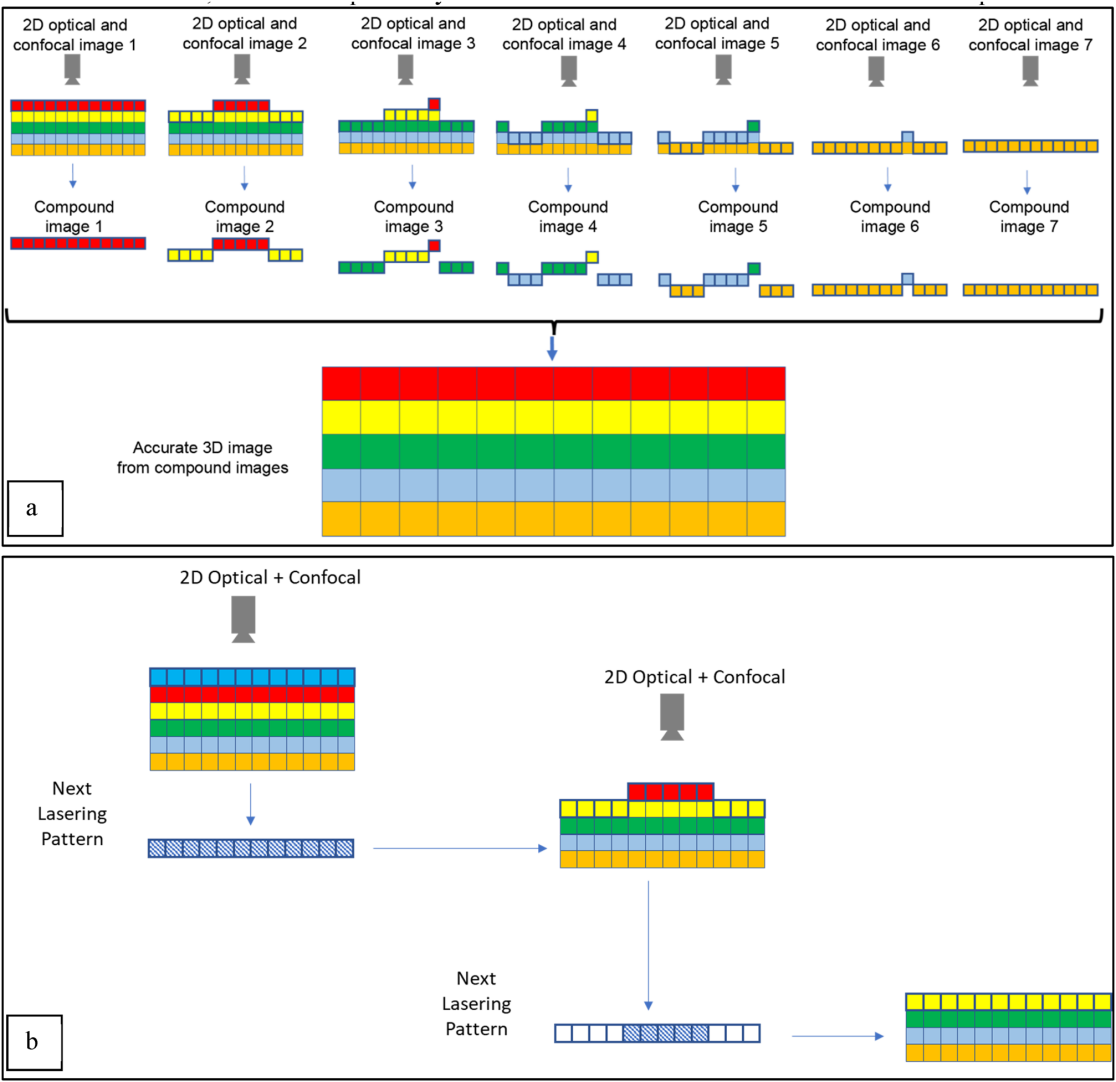
(**a**) Proposed approach for sampling the volume of interest addresses the distortion issue of the resulting 3D image; (**b**) Laser cutting pattern is determined based on the height profile to maintain the height variation within a certain limit.

For acquiring a 3D image of the sample, 3D points must be sampled from the volume of interest. Figure 1 demonstrates the main challenge associated with the conventional approach for acquiring the 3D image of a sample from the images of the exposed layers. A key assumption of the conventional approach, as mentioned earlier, is that the exposed layers are flat and thus the 3D information about each point can be obtained from the corresponding 2D image and knowledge of the layer thickness. However, if for any reason, the exposed layers deviate from being flat, the use of this approach will result in a distorted 3D image which will introduce difficulties and inaccuracies in the follow-up analysis efforts. In the proposed approach, instead of acquiring a 2D image of the exposed surface, we will acquire a 3D surface image by combining the optical image and the corresponding height map of the surface. In this work, such a height map is obtained by leveraging confocal microscopy. Figure 2a schematically demonstrates the proposed approach.

### Workflow overview

The proposed method is based on conducting consecutive optical and confocal imaging and laser delayering repeats. In each repeat, first, an optical and confocal image of the sample is acquired. Based on the measured height at each region, it is decided whether that region must undergo lasering in that repeat. Then, the laser delayering at the required regions will be performed. The 3D volumetric image of the sample is constructed from the optical and confocal images obtained at the first step of each repeat (Fig. 2a, first row). At each point on the 2D $xy$ plane, the pixel color from the optical image is combined with the height information of that point (in the $z$ direction) obtained from the confocal height map, resulting in the compound image of the layer (Fig. 2a, second row). The compound image of the layer will be registered in a universal coordinate system and will be embedded in the 3D image (Fig. 2a, third row).

**Figure 3:**
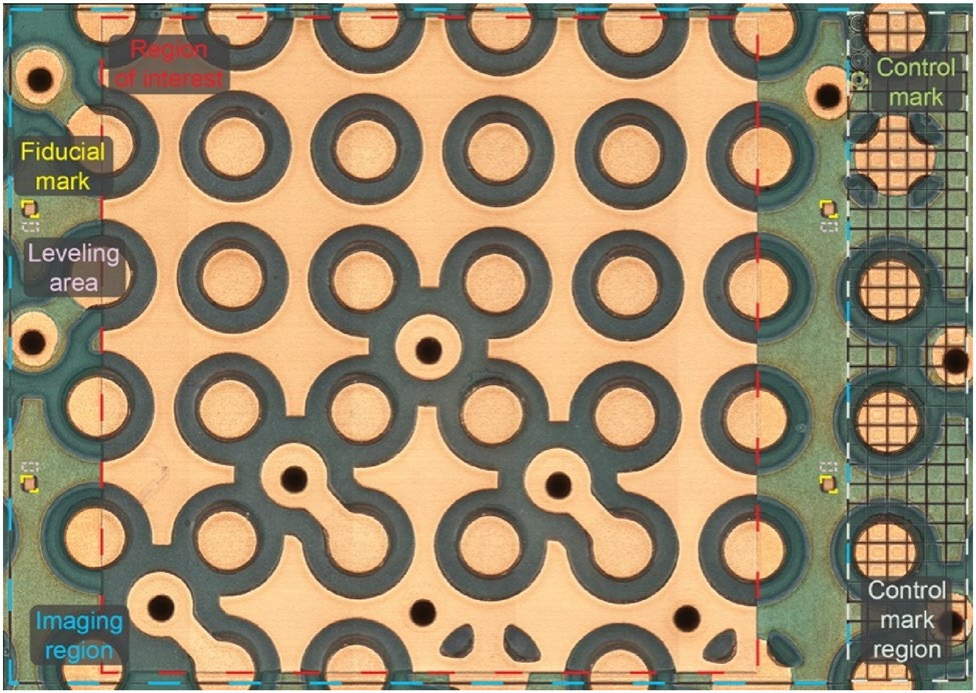
The different regions, marks, and areas on the sample for the whole process.

A set of fiducial marks (Fig. 3), that are created on the sample at the beginning of the process (i.e., before repeat #1), will aid in establishing and using a universal coordinate system (Fig. 2b). The centers of four square-shaped fiducial marks carved outside the region of interest serve as anchor points for translation, rotation, and tilting correction that may occur across images of different layers.

In each repeat, based on the height map of the region of interest, a mask will be generated that prescribes the upcoming lasering recipe. Only areas whose heights (as indicated by the height map) are larger than a certain threshold will be lasered in the lasering step of the upcoming repeat. Given that the ablation rate is not the same in different areas of the region of interest because of the presence of different materials, doing so is necessary to keep the layer height variation within a certain limit to allow the confocal microscope to be able to capture the entire region of interest (Fig. 2b). Masking will further speed up the overall process by shortening each confocal imaging step because to obtain the height profile of the surface of interest, the confocal microscope will obtain information at different height increments. The number of such increments, and thus the imaging time, is directly proportional to the height range of the region of interest, for a certain desired height resolution.

The outcome of the described workflow is a 3D image of the sample. The following outlines the detailed steps of the workflow.

1. The region of interest (ROI) is identified (Fig. 3), and an overview X-ray CT image is obtained for preliminary identification of the expected number of layers and their thickness. Note that obtaining the X-ray CT image is not a necessary task.
2. Four $100\,\mathrm{ \mu m}\times 100\,\mathrm{ \mu m}$ fiducial marks are created using the laser outside of the ROI (Fig. 3). These will be used for two purposes: (a) high precision alignment of the lasering pattern across different repeats; and (b) high precision registration of surface images towards obtaining a 3D image of the volume of interest.
3. Using the confocal microscope, optical and height images of the ROI and the fiducial marks are obtained (the imaging region in Fig. 3). Given that the size of the ROI is larger than the field of view of the microscope, a mosaic imaging strategy is adopted where the multiple imaging tiles are registered after image acquisition (Fig. 6a).
4. The obtained optical/height images are processed to produce the mask that will be used to perform the next lasering step. The following procedure will be adopted: (a) Based on thresholding on the depth (height), the fiducial marks are identified; (b) The centers of fiducial marks are utilized to correct the image for rotation and translation; (c) The image of a small intact area outside (above or below) each fiducial mark is analyzed and used to level the height map (the leveling areas in Fig. 3); and (d) Based on the height map of the ROI, a lasering mask is generated. The lasering mask determines which regions must be lasered and which regions must remain intact at each repeat.
5. Using the mask, the ROI is lasered.
6. Steps 3–5 are repeated until the desired volume is entirely captured.

In this process, a control region and control marks are also used (Fig. 3). This is not necessarily required, but they are used to control and check the manual alignment of the sample after bringing it back from the confocal microscopy to the lasering system for the next round of lasering.

### Sample

**Figure 4:**
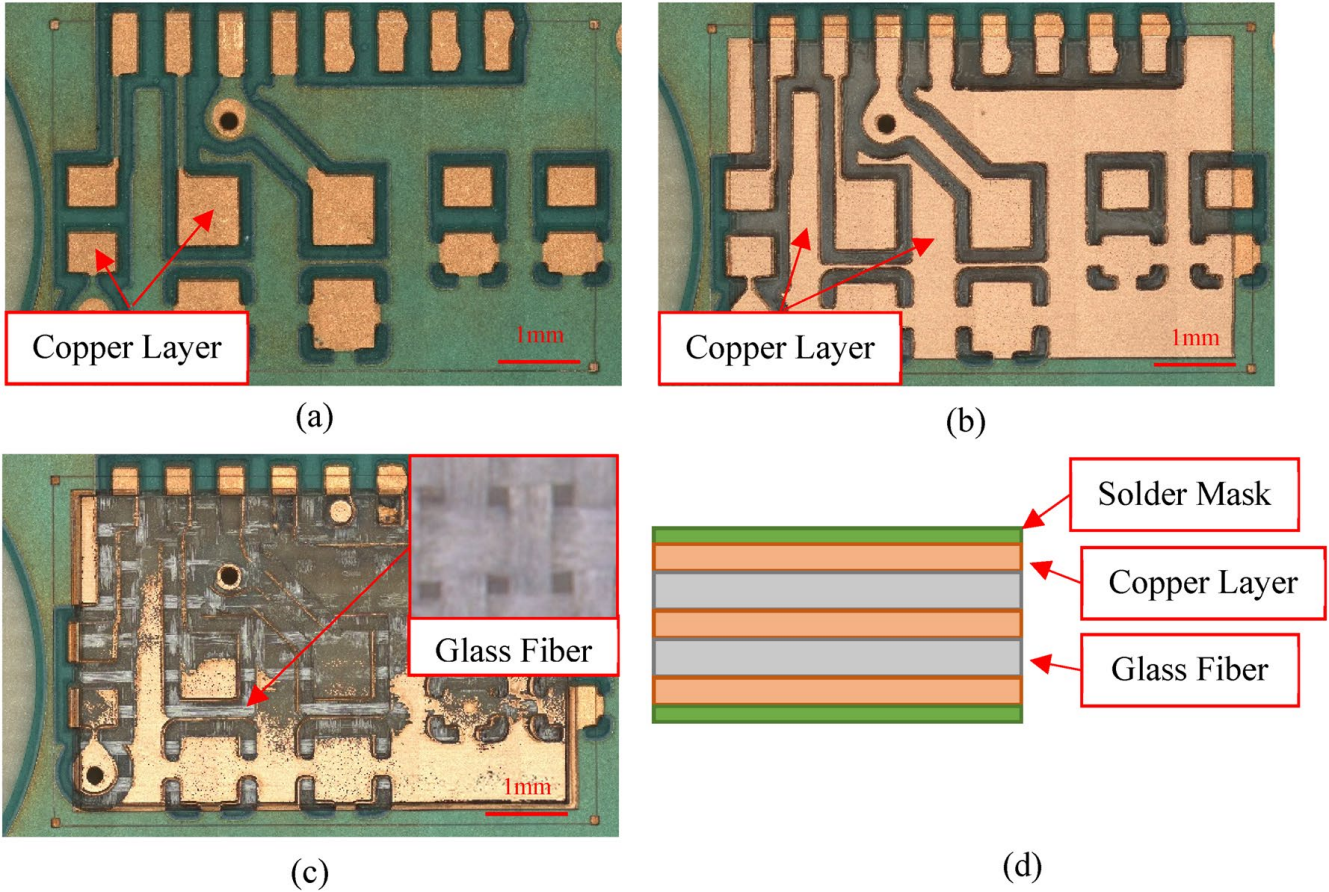
Digital image of the PCB board after: (**a**) 0 cycles of lasering; (**b**) few cycles of lasering; and (**c**) tens of cycles of lasering. (**d**) Schematic illustration of PCB cross section.

The sample studied in this work is a typical printed circuit board (PCB) board that is widely available in the market. This sample consists of (1) insulating materials such as solder mask, (2) copper layers laminated to the substrates, and (3) glass fiber layers. Figure 4 illustrates different layers of the PCB.

### Laser setup

**Figure 5:**
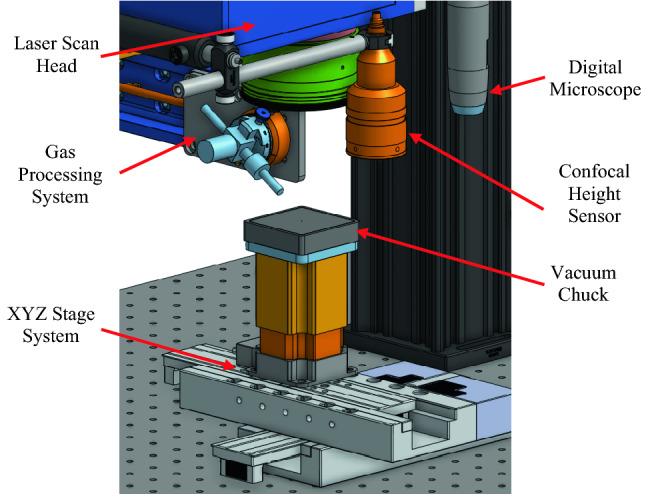
3D CAD design of the laser system.

Coherent Monaco 1035 nm 40 W laser (Coherent Monaco 1035-40-40, USA) with 252 fs pulse width that can produce a wide range of different pulse repetition rates, from single shots up to 50 MHz, was used in this study. The laser emits a 2.75 mm diameter beam that goes through a beam expander comprised of a fused silica 75 mm aspherical lens and a fused silica 300 mm convex lens to deliver a ~ 11 mm input beam diameter to a SCANLAB intelliSCAN_se_ 20 scanner that can provide a 2 m/s marking speed. The beam then goes through a telecentric fused silica F-Theta lens (TSL-1064-10-56Q-D20) with an effective focal length of 70 mm. The resulting theoretical spot size within the setup is ~ 8.5 µm. Computer-aided design (CAD) of the laser setup is shown in Fig. 5. The laser processing system consists of 6 major components: laser scan head, confocal height sensor, digital microscope, gas processing system, XYZ stage system, and vacuum chuck. With the integration of the gas cleaning/cooling system, redeposition and heat-affected zone (HAZ) are significantly reduced. Table 2 provides the description and the resolution for relevant major components of the setup.

**Table 2:** Description and resolution of major components of the laser system.

Equipment	Description	Resolution
Keyence CL-P070G	Laser confocal height sensor: responsible for measuring the height of the sample to ensure accurate laser focus plane	0.025 µm
SCANLAB IntelliSCAN_se_ 20	Laser scan head: utilizing two galvanometer mirrors to scan or raster laser across the sample surface	< 0.4 µrad repeatability 20bit positioning resolution
DinoLite AM73915MZT	Digital Microscope: sample targeting and quick inspection post lasering	2560 × 1920 5 Megapixels 7 µm pixels at 220×
Zaber X-LDA150A	XY stages: translation of sample from the sensor to scanner to vision system	0.2 µm
Zaber VSR40A	Z stage: translation of sample to adjust focus during or before lasering	0.09525 µm step size < 1 µm repeatability

### Imaging

The Keyence VK-X3100 laser confocal microscope is used to obtain the optical and height information of the surface. A 10× objective lens is used. The numerical aperture for the lens is 0.3, its working distance is $16.5\, \mathrm{mm}$, and its field of view can vary from $168\,\mathrm{ \mu m}\times 126\,\mathrm{ \mu m}$ to $1849\,\mathrm{ \mu m}\times 1386\,\mathrm{ \mu m}$. The largest field of view is when an optical zoom of 0.7× (expanding the field of view) is enabled. The resulting images in this work consist of $1024\times 768$ pixels. Each pixel captures an area of $1.38\,\mathrm{ \mu m}\times 1.38\,\mathrm{ \mu m}$ on the sample.

**Figure 6:**
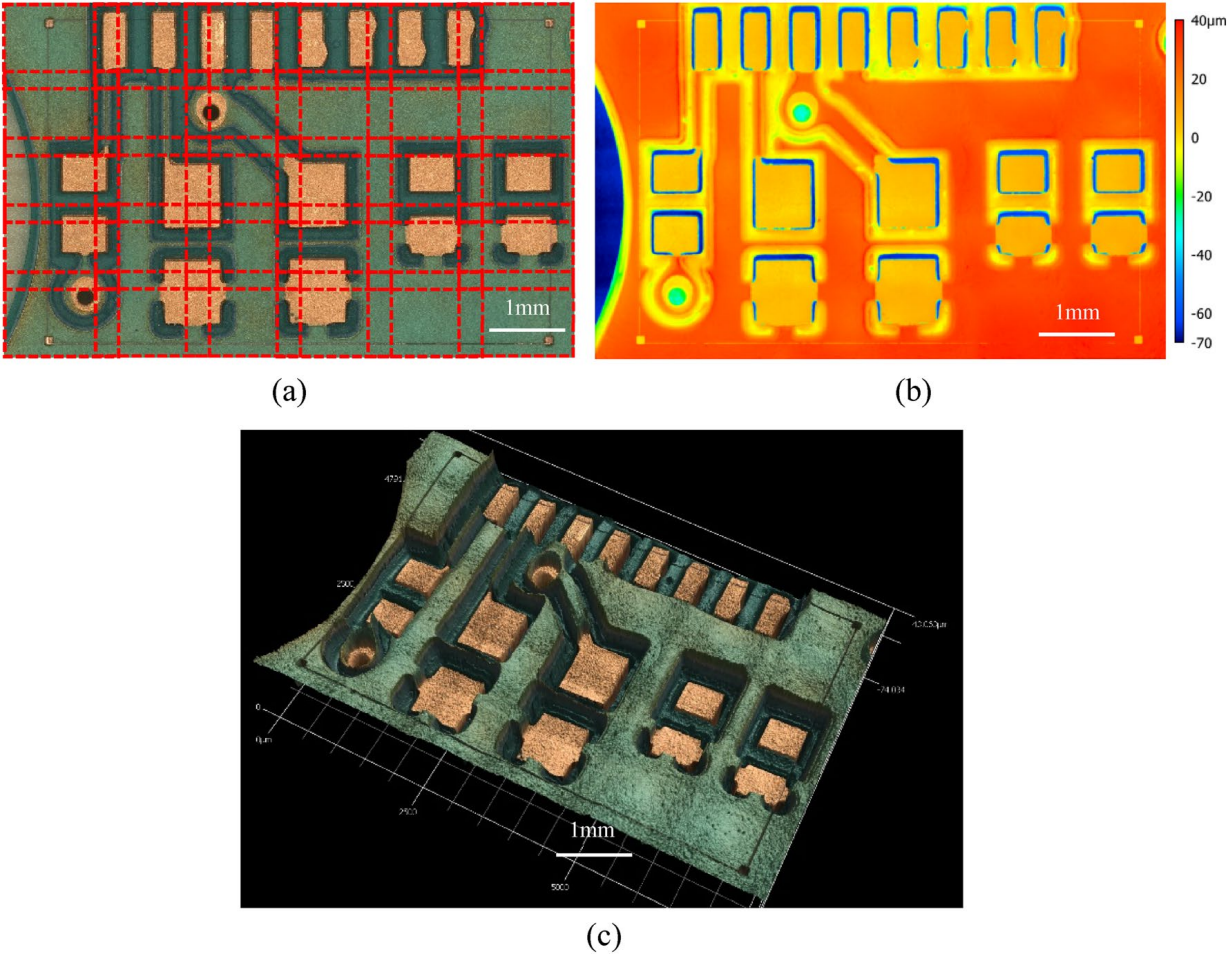
2D optical image with 6 × 5 imaging grid illustrated; (**b**) Surface height information of the same imaged area in (**a**), represented as a heat map; and (**c**) and 3D surface image obtained from fusing optical and confocal images.

The pitch in the $z$ direction (i.e., the relative motion of the microscope head with respect to the sample for acquiring information from different heights) is 2 µm. This specific combination of lens and pitch size is chosen to not only ensure the required data resolution but also to minimize the imaging time. To capture the entire ROI and the fiducial marks, a $6\times 5$ array of images is stitched together. This is illustrated in Fig. 6a. Per the described specifications, the total imaged area is about 7.6 mm $\times$ 4.8 mm. The total imaging time is about 5–30 min which varies based on the height range of the imaging region.

Note that the confocal height sensor (described in Table 2), which is a part of the integrated laser system and is used for the adjustment of the sample height to ensure in-focus lasering, is different from the confocal microscope used for creating the 3D surface height maps.

The used microscope, Keyence VK-X3100, provides three options for height measurement: (1) focus variation, (2) laser confocal, and (3) white light interference. In this work, the second option, namely, laser confocal, is used. In this method, the light emitted from a laser source is concentrated onto the object's surface via the confocal optical system. The concentrated light reflects off of the object's surface and returns to the photoreceptor through the same light path. Placing a pinhole on the way of the path to the photoreceptor, that must receive the light, ensures that no light other than what passes through the focal point of the objective lens can reach the photoreceptor.

The laser confocal system can precisely overlay optical (Fig. 6a) and confocal (Fig. 6b) images, resulting in a 3D surface image as depicted in Fig. 6c.

### Delayering using femtosecond laser

**Figure 7:**
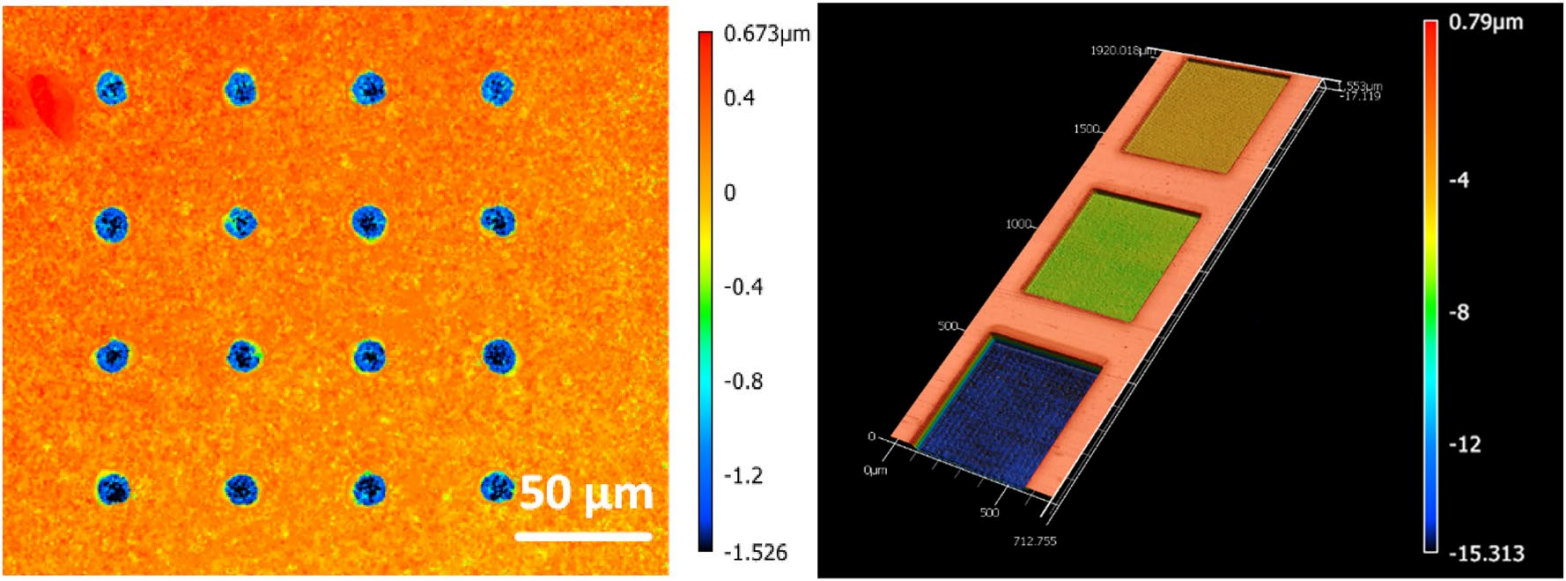
Laser optimization experimentation. Single-pulse experiments on plastic, for fluence and EPP investigations (left). Laser trenches on a copper substrate for repetition rate and overlap investigations (right).

The laser interacts differently with different materials. To achieve the best delayering results, proper lasering/scanning parameters must be selected based on the chemical composition of the PCB to increase throughput and to avoid unwanted phenomena such as melting that could otherwise impact the quality of the resulting 3D image. For the PCB sample, as mentioned earlier, the three main material compositions are copper, plastic, and glass fiber. A recipe-building process was conducted to find a single set of laser parameters that would be used to remove all three materials. The factors determining the selected recipe included material removal rate, material removal cleanness, size of HAZ, and precision of laser triggering/scanning to prevent laser dwelling. To optimize the lasering/scanning parameters for plastic, glass fiber, and copper, the effects of the lasering parameters were studied through single laser pulses as well as through the formation of rectangular trenches. In all three cases, confocal microscopy was utilized for imaging, followed by image processing and data analysis steps. Parameters that were optimized included energy per pulse (EPP), fluence, repetition rate, and pulse overlap in $x$ and $y$ directions. Figure 7 displays an example of the obtained data. The left part of Fig. 7 shows the single pulse experiments for plastic where fluence and EPP are optimized. The right part of the figure shows the trench experiments for copper, where the depth of cut, repetition rate, and overlap in x and y are optimized.

**Figure 8:**
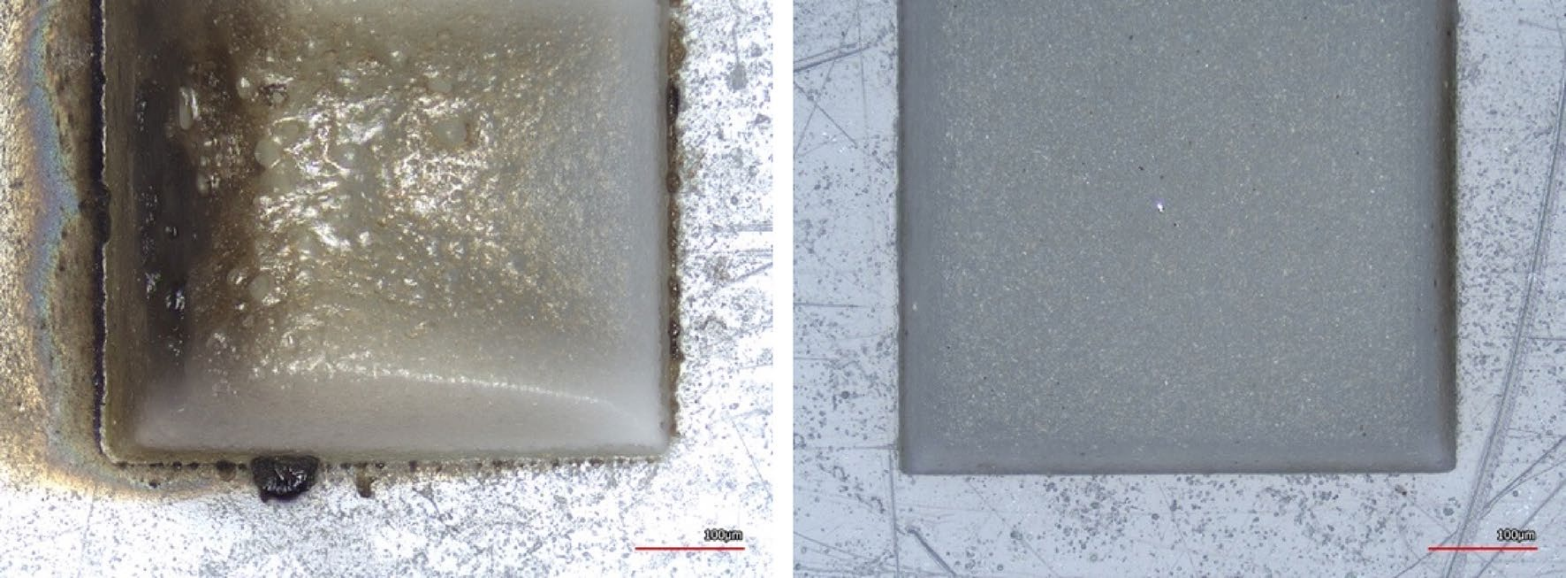
Different recipes ablating an ABS plastic sample. The left sample is lasered with an improper and the right one is lasered with a proper lasering recipe.

Table 3 summarizes the final selected parameters for lasering and the processing time per lasering cycle for a $6.4\,\mathrm{mm}\times 4\, \mathrm{mm}$ ROI. The used fluence is greater than the ablation fluence for both copper and the substrate. The ablation threshold fluence for copper is reported to be 0.35 J/cm^2^ for the 1035 nm wavelength^16^. The ablation threshold fluence for the SiO_2_ is reported to be 0.318 J/cm^2^^20^. For polyethylene terephthalate (PET) plastic, ablations fluences as low as 0.05 J/cm^2^ have been tried but the optimal threshold has been reported to be 0.4 J/cm^2^, below which solidification of the melt components after ablation leads to uncontrolled geometric changes in the shape of the micro-hole structures^21^. The optimization of parameters in this work has been conducted to prevent the distortion in the final 3D reconstructed image due to effects such as swelling and color change. The mitigation of HAZ to the extent that such aims are achieved is sufficient for the purpose of this work. Figure 8 compares an ABS plastic sample that is ablated with proper lasering parameters versus one that has undergone lasering with an improper recipe.

**Table 3:** The selected lasering parameters and the processing time per lasering cycle for a 6.4 mm × 4 mm region of interest (ROI).

Fluence (J/cm^2^)	Repetition rate (MHz)	Scan speed (m/s)	X-overlap (%)	Y-overlap (%)	Time per cycle (6.2 mm × 4 mm)
3.12	0.1	0.375	50	50	20 s

### The tradeoff between ablation rate and cleanliness of the delayering process

**Figure 9:**
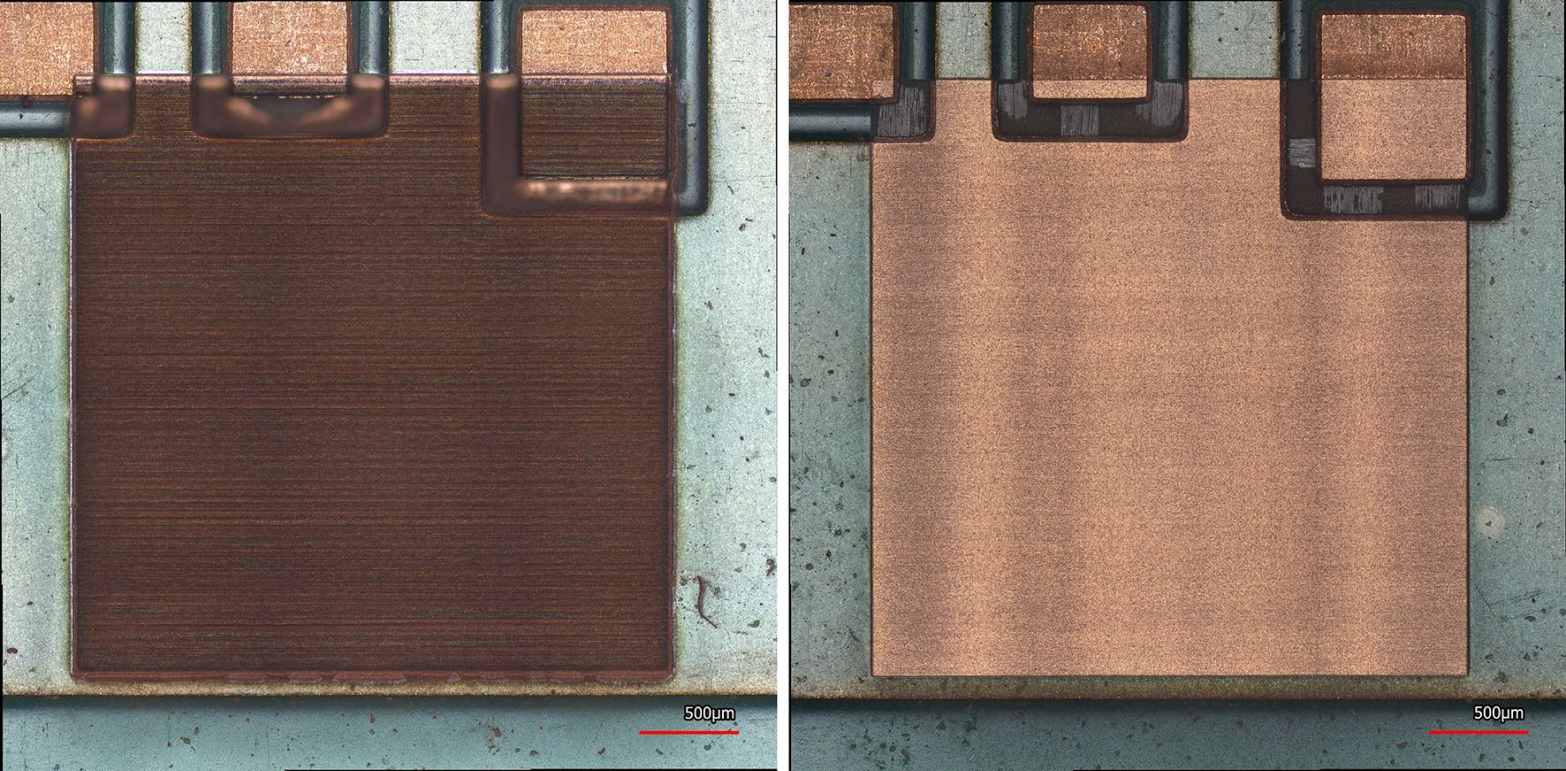
The tradeoff between throughput and cleanliness is shown. Left: use of high power to create a trench. Right: use of low power and a higher number of cycles to obtain a trench with the same depth.

As a part of the parameter optimization process, it also has been attempted to eliminate lasering artifacts such as darkening. There is a tradeoff between the laser ablation rate and the cleanliness of the delayering process, and the parameters used for laser delayering in this work are selected based on a parameter optimization. Here, a key parameter is the used energy per pulse (EPP). High values for EPP result in high ablation rates, but the surface quality and thus the accuracy of the final reconstructed 3D image will be poor. Figure 9 demonstrates the tradeoff between the throughput and quality.

### Dwelling compensation

**Figure 10:**
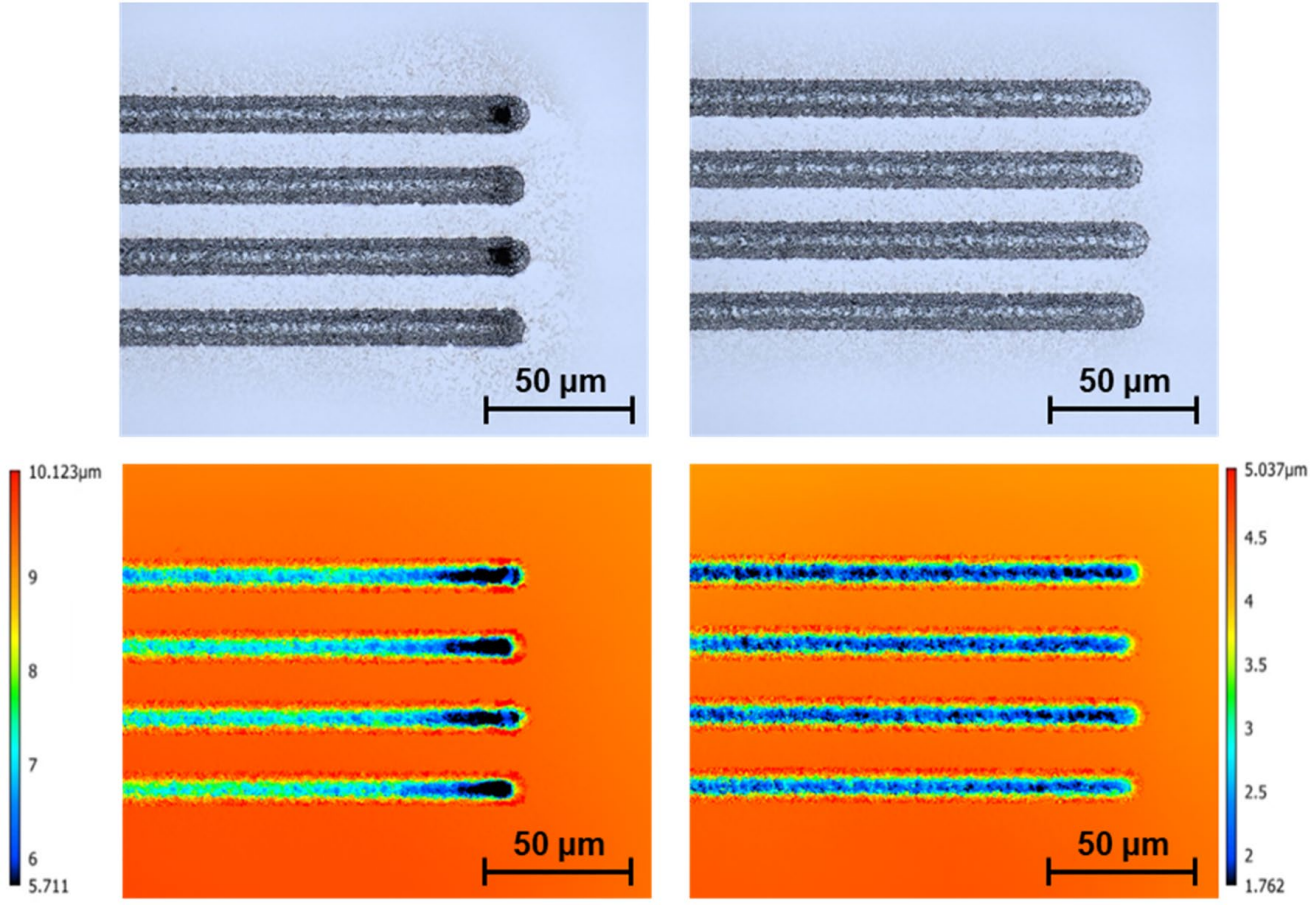
Laser lines drawn on silicon with no dwelling compensation depicting “burn-in” and causing uneven milling at the edges (left), and an example of lasering with dwelling compensation (right).

**Figure 11:**
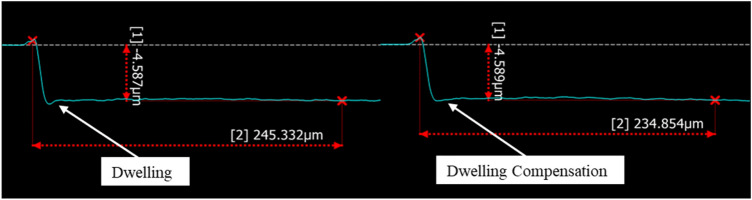
An example of dwelling caused by scanner mirror acceleration (left). Dwelling compensation utilizing a developed intelligent scanning system (right).

While attempting to precisely delayer material, dwelling artifacts, which are caused by the tracking error of the scan head, must be considered. Tracking errors can be caused by various reasons such as the acceleration/deceleration time of the scanning mirrors and the communication time with the controller. Figures 10 and 11, respectively depict top/down and cross sectional views of this artifact before and after correction. The correction has been conducted utilizing an in-house algorithm that takes into consideration the size of the shape, which is being marked, the desired marking speed, the repetition rate of the laser, and the acceleration/deceleration of the mirrors. The algorithm generates precise delays in the order of μs that are applied for the on/off triggering of the laser. The delays effectively ensure that at no time during marking, the overlap of pulses is more than specified. This is a necessity in precise delayering as the presence of dwelling will lead to increased HAZ and depth of cut, and obscuring features at a μm scale. There is an alternative solution provided directly through SCANLAB scanner systems known as skywriting. Skywriting adds a run-in and run-off area to allow the mirrors to pre-accelerate and post-decelerate for every line that is lasered, ensuring constant speed for the specified lines. There are, however, a few drawbacks to skywriting: it is a proprietary solution that is offered only for the SCANLAB scanner; setting the delay parameters needs optimization for a specific combination of laser and scanning parameters, and in most cases, it adds about 30–50% to the processing time. Having said that, the developed algorithm and the skywriting method can be interchangeably used to eliminate dwelling issues.

### Gas cleaning/cooling

**Figure 12:**
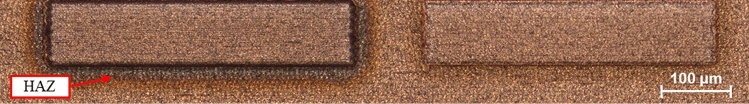
Comparison of laser processing of copper without gas processing (left) and with gas processing (right).

In this work, the snow cleaning method is used to clean the surface after each lasering cycle to maintain optimal laser ablation by preventing the redeposition from interfering with the next lasering cycle and welding back onto the sample^22^. The snow cleaning uses a combination of dry ice particles and gaseous phase to effectively remove particles that are as small as $0.02\, \mu \mathrm{m}$. The gas is fed into the nozzle, and the small orifice on the solenoid unit allows a controlled expansion of high-pressure gas to atmospheric pressure accompanied by a large pressure drop that creates small dry ice particles, referred to as “snow”. We hypothesize that this process helps with mitigating heat-affected zones (HAZs) in two ways: (1) removing the redeposited material from the lasered surface using gas injection prevents these particles from melting onto the surface during the next lasering cycle. We hypothesize that such a melting phenomenon is likely to happen because the redeposition will be at a different height than the rest of the surface, causing out-of-focus laser/matter interaction, which can lead to various heat-induced artifacts including melting. Such artifacts are manifested more significantly after a few cycles of lasering when no use of gas cleaning will result in piles of redeposited material on different regions of the sample; (2) due to the low temperature of the incoming gas (near the triple-point), it can cool down the sample, potentially contributing to the mitigation of HAZs. For the objective of this work, HAZ should be avoided only to an extent that it does not create issues at the optical imaging step. The optimization procedure to arrive at the right set of lasering/scanning parameters and gas injection has taken this measure into account. For each trial set of parameters, optical and confocal images are acquired for analysis of the resulting lasered surface. Application of gas cleaning/cooling also enabled us to obtain deeper trenches and cleaner walls. The necessity of applying this technology for obtaining high fidelity delayering in our work is showcased in Fig. 12, comparing the effects of the presence and absence of gas cooling on HAZ, processing copper.

### Image registration

The image data obtained during the delayering process must be registered, aligned, and leveled, to produce an accurate three-dimensional dataset. To accomplish this, four fiducial marks, in the shape of $100\, \mu {\text{m}}\times 100\, \mu {\text{m}}$ squares, are lasered on the area surrounding the to-be-delayered ROI (Fig. 3). After locating the fiducial marks by applying thresholding on the acquired height map, the $x$ and $y$ coordinates of the center of each square are extracted. Three of these centers are used for the correction of translation and rotation for registration of the image in a universal coordinate system. The fourth one will be used as a cross-check.

To align the height maps in the vertical direction (i.e., the $z$ direction, perpendicular to the $xy$ plane of the optical image), also a tilt correction should be performed. For this purpose, certain non-lasered areas in the surroundings of the fiducial marks will be used (leveling area in Fig. 3). The intact areas around three out of four squares will be used for leveling by attempting to bring them all to level 0. The intact area around the fourth square will be used for assessing the performance of the described process.

### Masking

**Figure 13:**
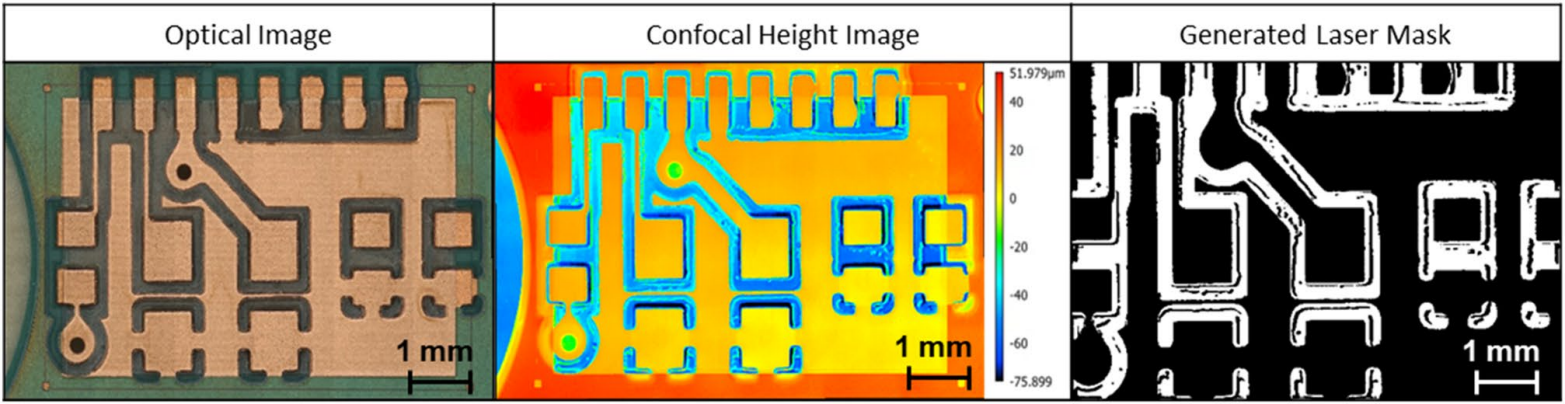
Generation of mask to be utilized during laser processing.

As mentioned earlier, samples composed of different materials, such as the PCB studied herein, experience different material removal rates at different regions, per the same lasering parameters. This will result in height variations across the region of interest. Although, thanks to the height information provided by the confocal images, this, in principle, will not affect the quality of the final reconstructed 3D image, given the limitations of height range in a confocal image, sample surface height variation must be kept within certain limits. Doing so will also help with cases where there is a limit on the allowed depth of cut for the entire sample, for example, when the sample must be used for a follow-up study afterward. To achieve this goal, a masking process is utilized, where at each repeat, based on the acquired height map, the lasering plan is programmed. Such a plan will indicate which regions must be lasered, and the rest will be skipped. More elaborately, by applying the 2-means algorithm on the heights, the different areas of the region of interest are partitioned into “low” and “high” classes. At each lasering step, only the “high” areas are lasered. Consequently, all areas of the region of interest remain within a relatively small depth band during the entire process. Figure 13 displays a generated mask for the upcoming lasering step. The black areas are the ones being lasered.

### 3D reconstruction

After acquiring all the images, the image registration process is applied to generate aligned sets of optical/height images. Doing so will result in a set of optical images for which the height of each pixel is known. The lower and upper limits of height, across all pixels present in the dataset, determine the height range of the tomographic 3D image. Importantly, since the heights of pixels of the acquired optical images are known, such information can be used to produce the corresponding voxels of the three-dimensional reconstruction image. For each voxel, two forms of information, namely the color and the material composition information can be assigned. The color information is acquired optically, and the material composition information is deduced from the ablation rate information as described in the following section. Further, a vertical linear interpolation between the known voxels provides an estimation of the remaining voxels between the known ones. It is important to note that, like the conventional volumetric imaging with flat layers, the accuracy of the final 3D image is controlled by the layer thickness at each step of delayering, which is controllable by lasering parameters.

### Resolution

The resolution of the resulting 3D reconstructed image must be studied from two aspects, namely lateral and vertical. The lateral resolution of the 3D reconstructed image is determined by the optical imaging settings. In this work, the lateral resolution is $1.38\, \mu \mathrm{m}$. This number can be significantly improved by changing the imaging settings at the cost of longer imaging times. The vertical resolution of the 3D reconstructed image, however, is determined by two factors: (1) the laser delayering resolution, which specifies the thickness of the removed layers, and (2) the vertical resolution of the height map acquired by the confocal microscope. In this work, the same set of lasering and scanning parameters is used for all the laser ablation processes. For the set of parameters used, the removal rate is $5.7\, \mu \mathrm{m}/\mathrm{cycle}$ for copper and $36.1\, \mu \mathrm{m}/\mathrm{cycle}$ for glass fiber. This number can be significantly reduced by using a different set of laser parameters, e.g., one with lower EPP, at the cost of longer overall process times. The vertical resolution of the confocal height map is determined by a variable so-called as the pitch value which in this work has been selected to be $2\, \mu \mathrm{m}$. Again, a lower value can be selected for this parameter at the cost of longer imaging times.

### Material characterization and segmentation

We previously proposed a material detection approach based on lasering parameters and the surface parameters including depth of cut, roughness, and skewness of the lasered area^23^. Further, the results of the new experiments reveal that the ablation rate can merely distinguish between plastic and copper in the PCB sample. At every round of lasering, an area in the region of interest undergoes lasering if it is present in the lasering mask. Therefore, at each repeat, for each lasered area, the number of cycles this area was lasered as well as the lasering parameters are known. Using the information of the height difference (before and after lasering) and the number of lasering cycles, the material for every lasered area is predicted. In this process, the entire volume is divided into sub-volumes, each consisting of a group of voxels. Each sub-volume is assigned a label, indicating its material composition. Given that due to height variations, lasering artifacts, and lighting conditions, the same type of material may look slightly different in different regions of the sample, the material prediction using the proposed method will help establish a much higher quality 3D image. Importantly, such an image is also inherently segmented, negating the need for labor-intensive manual segmentation efforts.

In this work, the ablation rate has been used as the sole parameter for differentiating materials. Therefore, the ability of this method in material differentiation depends on the detectable height difference between different materials as they undergo laser processing, which in turn is determined by the vertical resolution of the confocal microscope. We previously demonstrated that considering other surface metrics, such as roughness and skewness in conjunction with the ablation rate can improve the differentiation process^23^. To this date, we have demonstrated that copper, aluminum, silicon, and plastic can be easily distinguished using this method due to their different interaction with the laser. A study on a wider range of materials is planned for future work. It is worth emphasizing that since the proposed segmentation method uses only the height information obtained from the confocal microscope to differentiate between different materials, the color information, acquired by the optical microscope, will not affect the accuracy of the produced result regarding the material segmentation.

## Results and discussion

The results of applying the proposed method on a PCB for acquiring an image with a total height range of about 700 µm are provided. The entire process took about 20–30 h to complete. Table 4 juxtaposes several selected $xy$-plane sections of the resulting PCB 3D image with the corresponding X-ray CT images. As can be seen, the resulting 3D image obtained by applying the proposed method is richer in terms of information content. First, all the information that can be deduced from the X-ray CT image is also visible in the optical/confocal 3D image. Further, the resulting optical/confocal 3D image carries color information, helping distinguish different material compositions. Finally, the high-resolution optical information from the glass fiber material that is provided using the proposed method, is absent from the X-ray CT images.

**Table 4:** Some $xy$-plane sections of the resulting PCB 3D image using the proposed method and the corresponding X-ray CT images.

The $xy$-plane section of the resulting 3D image	The corresponding X-ray CT image
	
	
	
	
	

**Figure 14:**
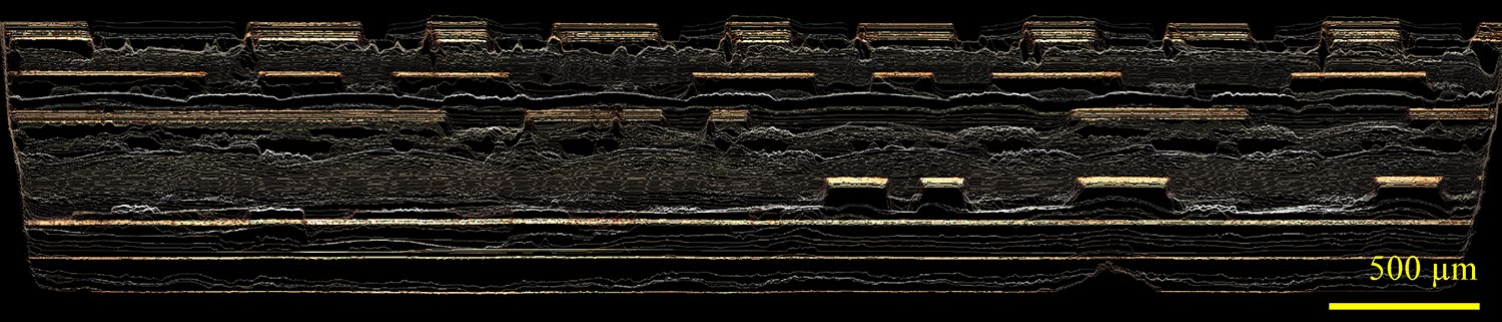
Cross sectional view of the volumetric image data as collected by the confocal microscope at different layers throughout the consecutive delayering/imaging procedure. The aspect ratio is 1:1.

Figure 14 shows a cross-sectional view of the volumetric image data as collected by the confocal microscope at different layers throughout the consecutive delayering/imaging procedure.

**Figure 15:**
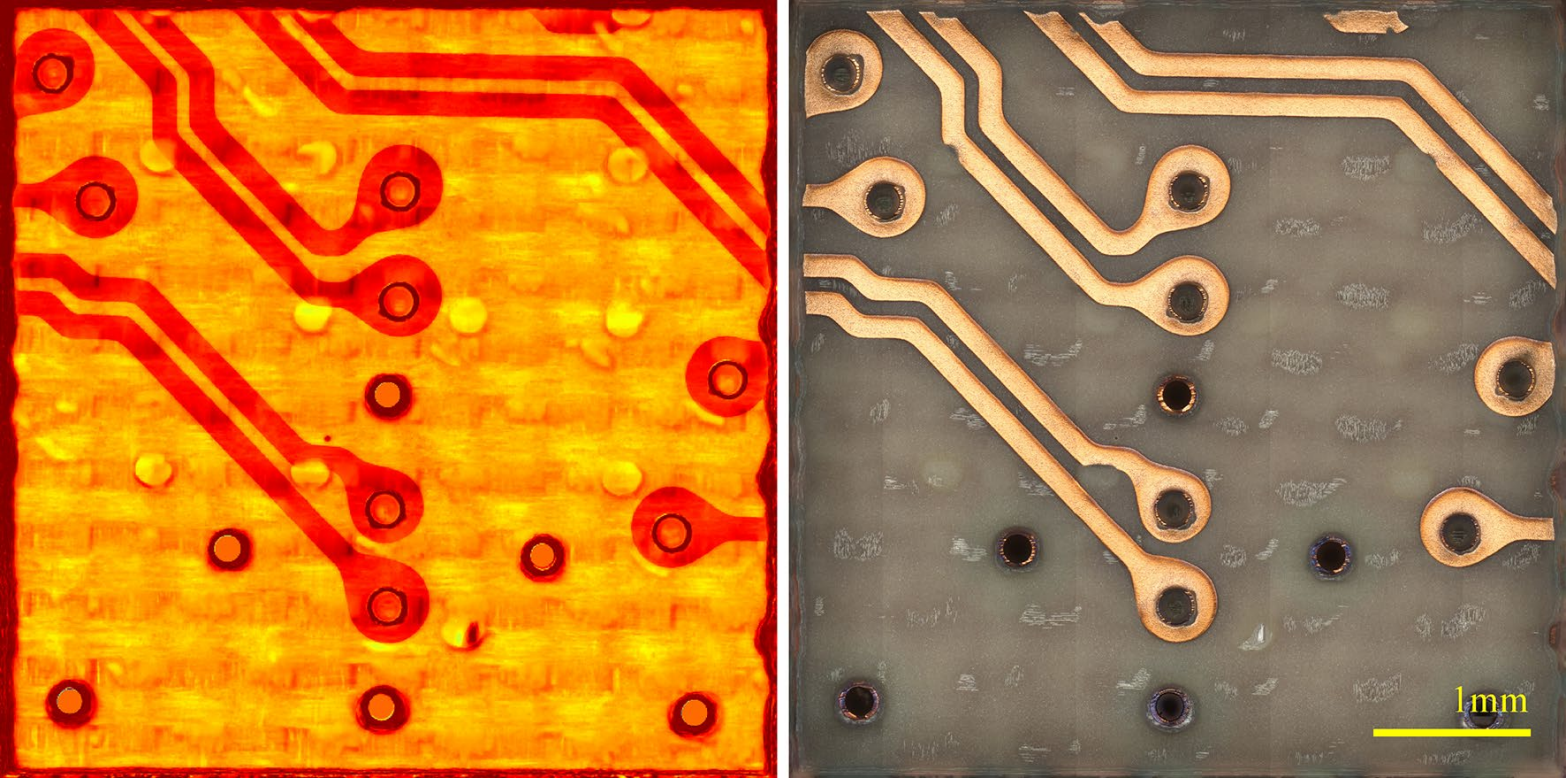
Automated image segmentation using the ablation rate information. Left: A heat map of the ablation rate (red is low, and yellow is high). Right: The optical image of the same region.

Figure 15 showcases the capability of the method for segmenting the resulting 3D image based on the ablation rate information. The left image of Fig. 15 is a heat map of the ablation rate, where red and yellow correspond to low and high ablation rates respectively. It must be emphasized that for obtaining this image no information from the optical images has been used. The left image shows the optical image of the same region.

## Conclusion

In this work, we proposed a novel method and a workflow for obtaining high-resolution 3D images of samples using femtosecond laser ablation for delayering and optical and confocal microscopy for imaging. Importantly use of confocal microscopy addressed the challenge of non-flat layers that arise due to the differential ablations across different materials. The proposed method outperforms X-ray CT imaging in terms of resolution and information content and is orders of magnitude faster than FIB/SEM.

## Data Availability

All the data generated or analyzed during this study are included in this published article.
